# Community development, implementation, and assessment of a NIBLSE bioinformatics sequence similarity learning resource

**DOI:** 10.1371/journal.pone.0257404

**Published:** 2021-09-10

**Authors:** Adam J. Kleinschmit, Elizabeth F. Ryder, Jacob L. Kerby, Barbara Murdoch, Sam Donovan, Nealy F. Grandgenett, Rachel E. Cook, Chamindika Siriwardana, William Morgan, Mark Pauley, Anne Rosenwald, Eric Triplett, William Tapprich

**Affiliations:** 1 Department of Natural and Applied Sciences, University of Dubuque, Dubuque, Iowa, United States of America; 2 Department of Biology and Biotechnology, Worcester Polytechnic Institute, Worcester, Massachusetts, United States of America; 3 Department of Biology, University of South Dakota, Vermillion, South Dakota, United States of America; 4 Department of Biology, Eastern Connecticut State University, Willimantic, Connecticut, United States of America; 5 Department of Biological Sciences, University of Pittsburgh, Pittsburgh, Pennsylvania, United States of America; 6 Department of Teacher Education, University of Nebraska at Omaha, Omaha, Nebraska, United States of America; 7 Department of Biology, Fairmont State University, Fairmont, West Virginia, United States of America; 8 Department of Science and Mathematics, Texas A&M University - Central Texas, Killeen, Texas, United States of America; 9 Department of Biology, College of Wooster, Wooster, Ohio, United States of America; 10 Division of Undergraduate Education, Directorate for Education and Human Resources, National Science Foundation, Alexandria, Virginia, United States of America; 11 Department of Biology, Georgetown University, Washington, DC, United States of America; 12 Department of Microbiology and Cell Science, University of Florida, Gainesville, Florida, United States of America; 13 Department of Biology, University of Nebraska at Omaha, Omaha, Nebraska, United States of America; Universidade de Mogi das Cruzes, BRAZIL

## Abstract

As powerful computational tools and ‘big data’ transform the biological sciences, bioinformatics training is becoming necessary to prepare the next generation of life scientists. Furthermore, because the tools and resources employed in bioinformatics are constantly evolving, bioinformatics learning materials must be continuously improved. In addition, these learning materials need to move beyond today’s typical step-by-step guides to promote deeper conceptual understanding by students. One of the goals of the Network for Integrating Bioinformatics into Life Sciences Education (NIBSLE) is to create, curate, disseminate, and assess appropriate open-access bioinformatics learning resources. Here we describe the evolution, integration, and assessment of a learning resource that explores essential concepts of biological sequence similarity. Pre/post student assessment data from diverse life science courses show significant learning gains. These results indicate that the learning resource is a beneficial educational product for the integration of bioinformatics across curricula.

## Introduction

### Integrating bioinformatics into the life science classroom

Life science research is in the midst of a paradigm shift, focusing more on interdisciplinary efforts that use streamlined high-throughput automation to generate ‘big data.’ Analysis of these data sets requires bioinformatics knowledge and techniques [1–3]. In addition, the importance of core competencies central to bioinformatics, including quantitative reasoning and the ability to tap into the interdisciplinary nature of science, is highlighted in the AAAS 2011 *Vision and Change* Report [4]. Thus, bioinformatics is becoming a critical part of the life scientist’s toolkit.

Efforts to establish bioinformatics core competencies and/or curriculum recommendations for undergraduate programs are described in the literature [5–8]. However, the pace of introducing bioinformatics concepts and tools into the undergraduate biology curriculum lags far behind what is needed for students to gain the skills required for advanced study and careers within the life sciences [9–11]. A commonly cited barrier to integrating bioinformatics into life sciences instruction is the lack of accessible ‘plug-and-play’ or easily adaptable materials that provide an intriguing ’hook’ to engage students [12]. In addition, biology instructors often lack training in bioinformatics and are thus not comfortable teaching it [13–15]. Thus, if they do implement an activity that uses a bioinformatics tool, little explanation is provided as to how the underlying algorithm works or what its assumptions are [11], knowledge that is critical for appropriately applying and using the tool. A central goal of The Network for Integrating Bioinformatics into Life Science Education (NIBLSE) is to address these barriers by developing, assessing, curating, and disseminating up-to-date and user-friendly open-access bioinformatics resources [16].

### The sequence similarity learning resource

Here we review the development, implementation, and assessment of an introductory bioinformatics learning resource [17,18] that explores the concept of sequence similarity and its biological implications. The resource is designed to capture students’ interest by enabling them to work on a short independent project. In addition, the resource provides learners with a clear explanation of the function and limitations of three alignment and phylogenetic tree-building algorithms (i.e., BLAST, Multiple Sequence Alignment, Neighbor Joining). This ‘under-the-hood’ knowledge is essential for properly interpreting the output of the programs that implement the algorithms. Several adaptations of the learning resource are available that allow its easy insertion into a variety of different classes (e.g., plant physiology, developmental biology, virology; [17,19–21]).

The sequence similarity learning resource is composed of four modules (Table 1) that can be used independently or together depending on course learning goals [17]. The first three modules explore how biologists quantify nucleotide and protein sequence similarity, compare a sequence to those in a public database (e.g., GenBank), and create phylograms that convey evolutionary relationships. In the fourth module, students apply the skills and conceptual knowledge gained in the first three to investigate a biological question of their own choosing.

**Table 1:** Sequence similarity learning resource module descriptions.

Sequence Similarity Module Title	Module Description
Module 1: Similarity and Sequence Alignment	Students explore the meaning of sequence similarity and then investigate how similarity can be quantitatively compared between two similar length proteins using a Blocks Substitution Matrix (BLOSUM) scoring matrix. This core concept and competency has utility for biologists seeking to identify conserved blocks of sequence in homologous proteins that may have structural and functional importance and hint at evolutionary relationships between two sequences.
Module 2: Sequence Alignment to a Database of Sequences	Students find local regions of similarity between a query sequence and a database of subject sequences using the Basic Local Alignment Search Tool (BLAST) algorithm and develop a basic understanding of the algorithm through a manual scoring exercise. This core concept and competency has utility for biologists seeking to identify conserved blocks of nucleotide or protein sequence that may or may not necessarily be homologous, but share common domains (often reused by similar families of proteins) that may hint at structure and function of a protein and hint at evolutionary relationships between two sequences.
Module 3: Phylogenetic Analysis of Homologous Sequences	Students practice accessing text-based FASTA-formatted sequence information via the National Center for Biotechnology Information (NCBI) databases as they collect protein sequence data for a multiple sequence alignment for the generation of a phylogenetic tree. Students construct a small tree manually using the Neighbor Joining algorithm. This core concept and competency has utility for biologists seeking to identify conserved protein domains and key conserved amino acid residues associated with structure and function within a domain in addition to allowing for visual depiction of evolutionary relationships between three or more sequences.
Module 4: Inquiry-Based Investigation	Students apply concepts and competencies from Modules 1–3 to address an authentic biological question. Instructors or students may choose between three investigations involving (1) the evolution of alcohol metabolism in hominids, (2) the evolution of Zika virus, or (3) determining the likely causative agent of an equine corneal ulcer.

An initial version of the learning resource was refined in a NIBLSE Resource Incubator [12]. Incubators are low-cost, short-term, online communities that develop open educational resources (OERs). Once refined, it was included in the NIBLSE Learning Resource Collection [17], an online collection of bioinformatics learning materials, and described in a recent publication [18]. Subsequently, a Quantitative Undergraduate Biology Education and Synthesis (QUBES) Faculty Mentoring Network (FMN) [22,23] supported implementation and assessment of the learning resource across multiple institutions. Here we report our assessment results, which demonstrate that the learning modules yield measurable objective learning gains and positive changes in student perceptions of bioinformatics knowledge and skills across diverse classrooms.

## Materials and methods

The study was approved by the Adams State University and University of Dubuque Institutional Review Board. Written consent was obtained from all human research subjects in the study.

### Learning resource development and implementation

**Fig 1:**
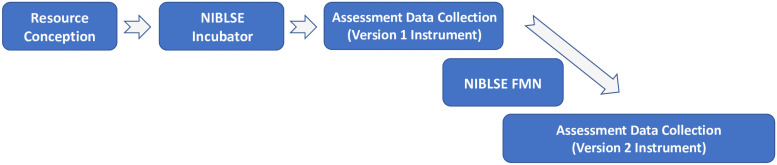
Development, implementation, and assessment of a NIBLSE OER learning resource. The original learning resource was conceived by a pair of institutional colleagues and implemented with course-specific student learning objectives. The resource was later expanded and targeted to a wider audience by a community of faculty through a NIBLSE Incubator. Following development of an assessment instrument, a NIBLSE Faculty Mentoring Network (FMN) recruited implementers and refined the assessment while collecting pilot assessment data. Data were collected from multiple institution and classroom settings concurrently during the FMN and after its conclusion. Vertically overlapping boxes indicate concurrent activities.

As previously stated, the focus of the resource is sequence similarity and its biological implications. It addresses Competencies 2, 4, 5, and 8 (S1 Table) of the NIBLSE Bioinformatics Core Competencies [7]. The resource was first used during the fall 2016 semester by a small group of faculty at a single institution aiming to integrate bioinformatics learning objectives in a general biology course (Fig 1). After piloting the resource at this institution, it was identified as a candidate for an Incubator [12], with the goal of providing a widely adaptable learning resource to integrate bioinformatics principles at the introductory level. The Incubator process generated a version of the resource with Creative Commons licensing and the QUBES Project [22] provided immediate public access within the NIBLSE Learning Resource Collection [17] for other educators, while acquiring input from diverse faculty within NIBLSE to validate the resource. The Incubator process further developed and enriched the content and facilitated the generation of supporting materials (e.g., teaching notes). In addition, the resource was converted into a modular format and expanded for a wider audience. After multiple pilot rounds of classroom implementation and refinement, a polished version of the resource was published in the journal *CourseSource* in 2019 [18].

The resource was further disseminated using a FMN in 2019 (Fig 1). In addition to implementing the resource in classrooms across the nation, a subset of FMN participants produced course- and learning goal-specific adaptations of the original resource, which are included in the Resource Collection [19–21]. The ability to update a resource and adapt it for multiple applications enables it to remain relevant in a rapidly changing field.

### Learning resource assessment

To test the effectiveness of the learning resource, a subset of FMN participants and NIBLSE network faculty administered a pre-/post-assessment instrument developed and refined during both the NIBLSE Incubator and FMN (Fig 1).

#### Student pre-/post-assessment instrument development

A goal of the Incubator was to design an assessment instrument to probe the effectiveness of the learning resource. Specifically, the goal was to measure both objective learning gains and individual student perceptions of learning, as the latter has been demonstrated to be important for persistence in STEM [24]. A community-based co-design process [25] was used to generate an initial version of the instrument, which used Likert-scale and rubric-scored open-response items (S1 Appendix) to measure the proportion of student participants who felt they had met, and objectively had met, the learning outcomes of the resource.

Based on the experience of administering the assessment and the resulting assessment data (S1 Text and S2 and S3 Tables), a second version (S2 Appendix), version 2, was iteratively developed as part of the QUBES FMN. To better quantify participants’ objective learning gains, version 2 converted post-instrument objective knowledge-based open-response to closed-response items, which were then featured in both the pre- and post-instrument. These replacement questions allowed for measurement of pre-/post-learning gains and, since they could be scored automatically, for the assessment instrument to be used with large numbers of participants. To reduce survey fatigue, the pre-assessment attitudes and perceptions questions were not included in the second version. Rather, this version incorporated retrospective perceptual items next to current perception statements to control for response-shift bias [26,27].

In its final form, version 2 of the instrument was a pre/post fifteen-item assessment consisting of a combination of multiple-choice and multiple-select questions. In addition, the post-assessment portion had a cluster of eight retrospective student perception questions based on learning outcomes. The perception questions were designed to measure perceptions of learning and used a 4-point Likert scale. Additionally, the instrument collected participant name, institution, and classroom instructor to facilitate matching pre- and post-assessments after final grades for the course were submitted. Version 2 of the assessment instrument was used for collecting all of the data reported in the results.

Instrument validity was established with respect to content validity [28,29], which was collaboratively established and affirmed by carefully mapping assessment questions to the content domain (S1 Table) and through systematic review by a panel of bioinformatics education experts associated with the Incubator, the FMN, and the wider NIBLSE Research Coordination Network [30,31].

Instrument reliability was examined by evaluating the internal consistency of individual items relative to the total post-assessment score (S4 Table). The procedure used was similar to the Kuder–Richardson Formula 20 (KR-20) and other reliability procedures [32], but was performed at the level of individual questions. This was done to investigate the contribution of each instrument item to the overall test score and to examine internal consistency more closely. Additional item analysis statistics included item difficulty, item discrimination, and point-biserial correlation index [33] to ensure that the test was well balanced and useful across the multiple classrooms taking the assessment. All of the questions were positively correlated with participant performance within a broad spectrum of item difficulty. That said, item #6 exhibited low point-biserial and discrimination indices suggesting a slight correlation based on holistic assessment performance and limited ability to discriminate (S4 Table). However, it was kept in the instrument so that the entire content domain is covered, with a suggestion to future users to modify the question for their own classroom contexts and curriculum [34,35].

In order to further explore reliability as related to internal consistency, Cronbach’s Alpha Reliability Analysis was performed on the scores of the post-assessment across institutions (n = 373 students). Cronbach’s Alpha is commonly used in reliability testing and compares the mean covariance between all test item pairs with the overall variance of test items, while adjusting for sample size. The statistic represents the interrelatedness of test items which should be high in an assessment that reliably represents a particular trait of interest. It is considered to be a relatively conservative test that often underestimates reliability [36,37]. The overall Cronbach’s Alpha level (ɑ) was computed at ɑ = 0.576, which would suggest slightly less than optimal reliability when using the instrument across the diverse set of institutions and courses. Further analysis showed that ɑ could be increased above the generally accepted 0.6 threshold by removal of two items that exhibited problematic discrimination (S1 Text and S5 Table), thus identifying items that instructors can further tailor to their classroom instruction.

#### Student pre-/post-assessment data collection

Administration of the pre-/post-assessment instrument with a diverse cohort of students (Table 2) undertaking the sequence similarity modules was completed during the spring 2019, fall 2019, and spring 2020 semesters. The assessment instrument was administered before and after completion of the modules. Assessment participant incentivization was at the instructor’s discretion and varied from presenting participation as a way to help improve future life science curricula to offering a nominal extra-credit opportunity. Data were collected electronically using a secure web-based platform either inside (Primarily Undergraduate Institution (PUI) General Biology, Developmental Biology, Molecular Biotechnology [spring 2019], Bioinformatics and Computational Biology) or outside (Research Intensive (RI) institution General Biology, Virology, Molecular Biology, Genetics, Molecular Biotechnology [spring 2020]) of class, based on the instructor’s discretion. With the exception of the spring 2020 cohort of Molecular Biotechnology students, all modules were done in the physical classroom. Due to a low response rate, assessment data from the Genetics course was not included in further analysis. When possible, the answers of the multiple-choice and multiple-select items were randomized. Matched pre-/post-assessment records (n = 373) were used for subsequent analysis. Assessment data were not accessed by the instructor of record until final grades were posted. All protocols were approved by the Adams State University (IRB #232017, #1122018, #3262019) and University of Dubuque (IRB#1031) Institutional Review Board (IRB) using a cross-institutional IRB application.

**Table 2:** The bioinformatics sequence similarity learning resource was implemented in a diverse set of courses across program-level and institution classification.

Course Content Focus*	Undergraduate Course Level†	Institution Classification‡
Bioinformatics and Computational Biology	100	RI
General Biology	100	RI
General Biology	200	PUI
Genetics	300	RI
Molecular Biotechnology	300	PUI
Molecular Biology of the Cell	300	RI
Developmental Biology	400	PUI
Virology	400	RI

*The General Biology course offered at the primarily undergraduate institution covered topics focused within the areas of cellular and molecular biology, while the General Biology course offered at the research-intensive institution focused on biological diversity and ecology.

^†^Within a 4-year undergraduate degree plan, 100–200 level courses are typically introductory in nature and require less prerequisite knowledge (OR fewer prerequisite courses). 300–400 level courses are typically advanced in nature and specialized in course content and typically reserved for upper-level students.

^‡^Research Intensive (RI) Institutions are doctoral degree granting universities with moderate to very high research activity. Primarily Undergraduate Institutions (PUIs) typically focus on conferring bachelor’s degrees, where the primary expectation for faculty is teaching, with research being a secondary focus.

#### Student pre-/post-assessment and perceptions data analysis

Pre-/post-assessment records were matched, with non-matching pre- or post-records removed prior to analysis. Of the 373 matched records, 11 records lacked complete Likert-scale student perceptions data and thus did not contribute to the student perceptual data analysis (n = 362). Multiple-select objective knowledge-based questions had two correct statements, which led to a scoring system that awarded 0.5 point for each correct selection and penalized -0.5 point for each distractor selected. The scores for each multiple-select question response item were summed to provide a single score for the question, which could be negative. The Cronbach’s Alpha instrument reliability metric was calculated using Statistical Package for the Social Sciences (SPSS), while item analysis was performed in Microsoft Excel. All other statistical analyses were performed using R (v. 3.5.1) [38]. Average learning gains per class were analyzed using one-sample t-tests with Benjamini-Hochberg correction [39]. A generalized linear model (GLM) was used to compare score difference (post—pre) among two factors: institution type (PUI vs. RI) and course type. A second model was used with the same factors but comparing only pre- scores to examine differences among groups prior to administration of the learning materials.

## Results

### Assessment of the sequence similarity learning resource

#### Learning gains were observed across the aggregate dataset

**Fig 2:**
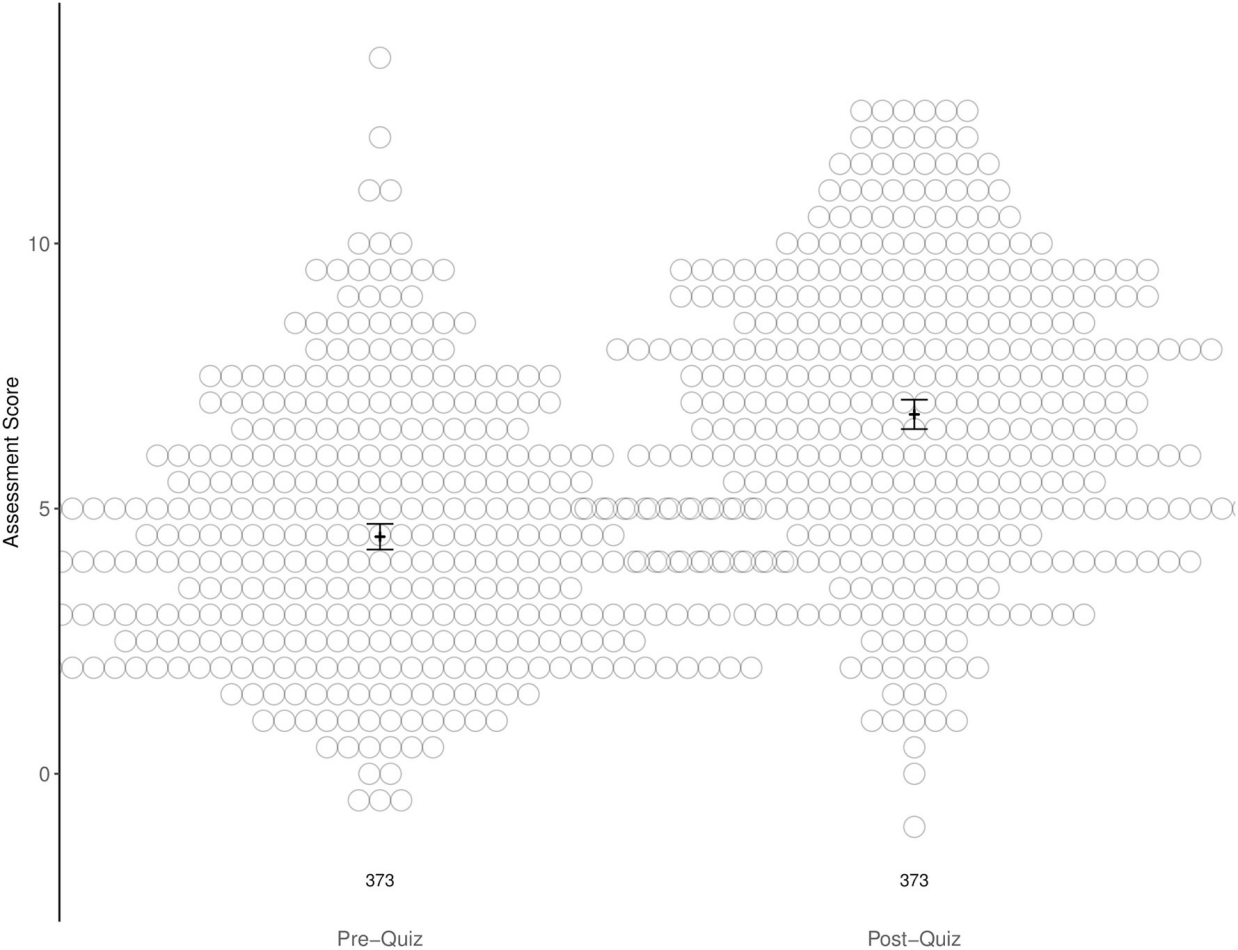
Aggregate pre-/post-assessment quiz scores indicate significant participant learning gains. The fifteen-item assessment consisting of a combination of multiple-choice and multiple-select questions was administered pre- and post-completion of the learning modules. Nine cohorts of student participants (n = 373) at independent institutions completed the assessment instrument with 7–28 days between pre- and post-assessment. Pre- (4.47) and post- (6.78) means are represented by a narrow black crossbar. The difference between the pre- and post-means has statistical significance (p < 0.00001, GLM). Black error bars represent the 95% confidence interval of the mean and the number of matched student assessment records is indicated below each swarm plot.

We investigated whether the set of fundamental sequence similarity learning modules could produce objective quantifiable student learning gains in diverse classrooms, from PUI to RI institutions, in varied life science subjects, and from introductory to advanced classes (Table 2). Participants were undergraduate students enrolled in life science courses taught by NIBLSE and FMN-participating faculty (Table 2). Our findings show that implementation of the modules led to objective student learning gains (Fig 2). Aggregate pre-/post-assessment scores exhibited a significant difference from 0 with an estimated mean increase of 2.31 (from 4.47 to 6.78 out of fifteen items, n = 373 subjects, p < 0.00001, GLM).

#### Learning gains were observed across diverse life science courses

**Fig 3:**
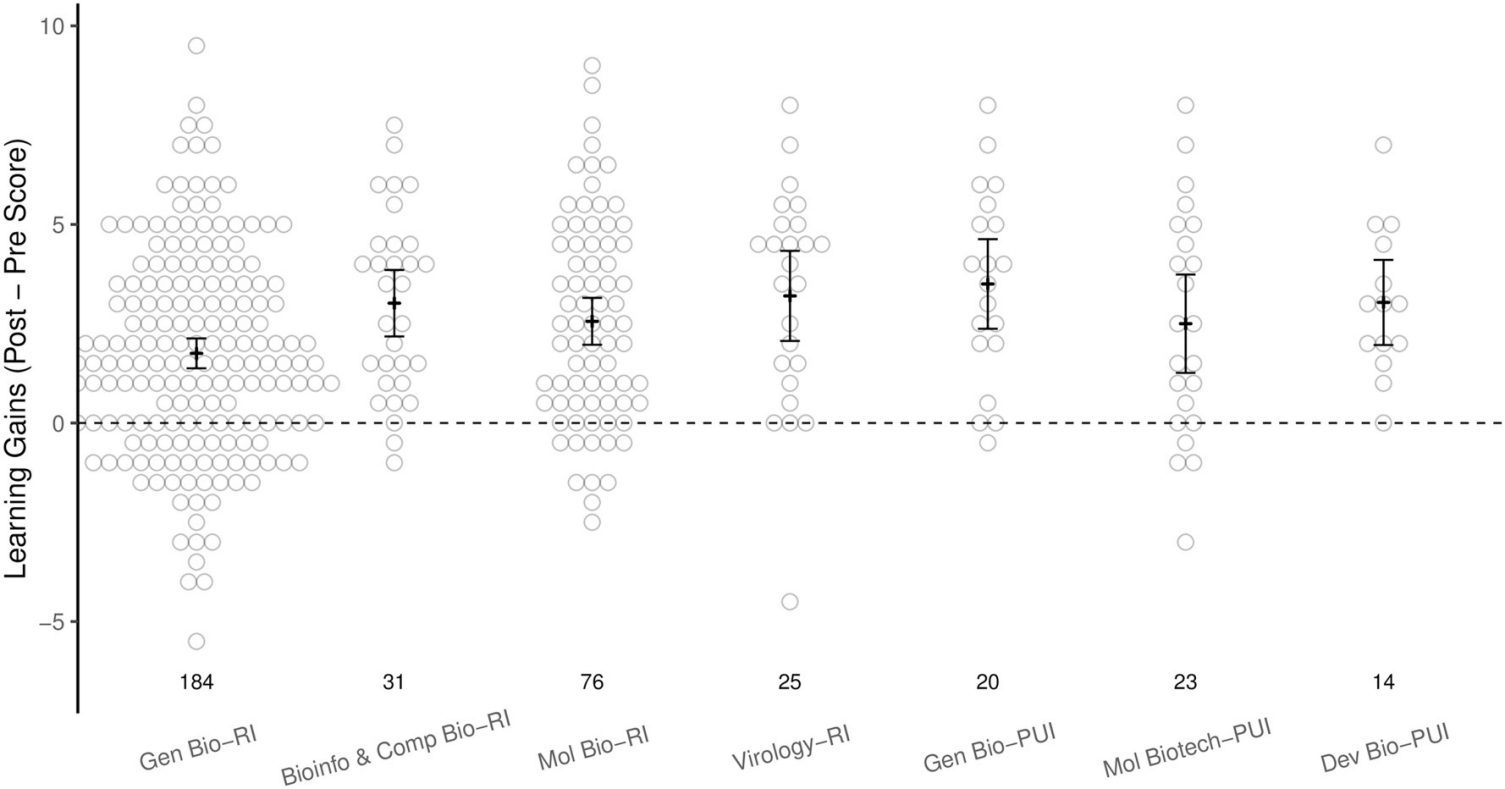
Learning gains from matched pre-/post-assessment quiz scores disaggregated by course type. Courses at PUIs in which the modules were implemented included General Biology, Molecular Biotechnology, and Developmental Biology. All others, including an additional General Biology course were at RI institutions. Means are represented by a narrow black crossbar. Black error bars represent the 95% confidence interval of the mean. The black dashed line indicates a pre-/post- difference of zero, indicating neither a learning gain nor loss. Learning gains significantly greater than 0 were observed in all classes (adj. p < 0.001, one-sample t-test). Sample size (n) for each course is shown above course name.

The versatility of the learning resource and its ability to be integrated across biology curricula is illustrated by the variety of courses in which it was implemented (Table 2). As shown in Fig 3, average learning gains were significantly above zero (0) for all courses (S6 Table, adj. p-value <0.001, one-sample t-tests with Benjamini-Hochberg correction).

We wanted to determine if quantifiable learning gains were independent of the course and institution type. In initial GLM testing, we included course type, institution type, and course levels as factors in the model to investigate whether objective quantifiable learning gains were present independent of these factors. As our design was not orthogonal, we found an expected high collinearity between course level and course type. Therefore, the final model used was a two-factor model with course type and institution type as factors.

This analysis found that there were significantly higher learning gains in courses taught at PUIs than those taught at RIs (S7 Table; p = 0.004, GLM), although the RI group included the largest class (RI General Biology), which showed the smallest learning gains. Similarly, the only significant difference in learning gains due to course type after accounting for institution type was found between RI General Biology and other RI classes. Thus, the observed difference in learning gains between institution types may have been confounded by class size, and in any case are small (Fig 3).

In analyzing pre-assessment scores we found no significant difference by institution type. However, there were several significant differences among the course types, with General Biology (RI) showing the lowest pre-scores (S7 Table). The other three courses taught at RI universities began at significantly higher pre-score levels than General Biology, while pre-scores in courses taught at PUIs were not significantly different from RI General Biology. Our general conclusion is that regardless of institution, course type, and initial knowledge level, all groups of students made significant learning gains through the use of the Sequence Similarity learning resource.

We noticed that the time spent completing the assessment was quite short (<4 minutes) for a significant percentage of tests, possibly indicative of students who were not fully engaged. When the student assessment dataset was filtered to remove these records (about 18% of overall scores), we observed an upward shift in average learning gains, as well as both pre- and post-scores, across the board (n = 306, a mean increase of 2.56 from 4.77 [pre] to 7.33 [post]). Interestingly, students from the General Biology (RI) cohort were notably a high percentage (>80%) of these short-duration submissions. Analysis of the filtered data indicated no statistically significant differences in learning gains between General Biology (RI) and the other courses, with the exception of the Bioinformatics course (S8 Table). In summary, we conclude that regardless of institution, class type, and initial knowledge level, all groups of students exhibited significant learning gains.

#### Student participants self-report perceived learning gains

**Fig 4:**
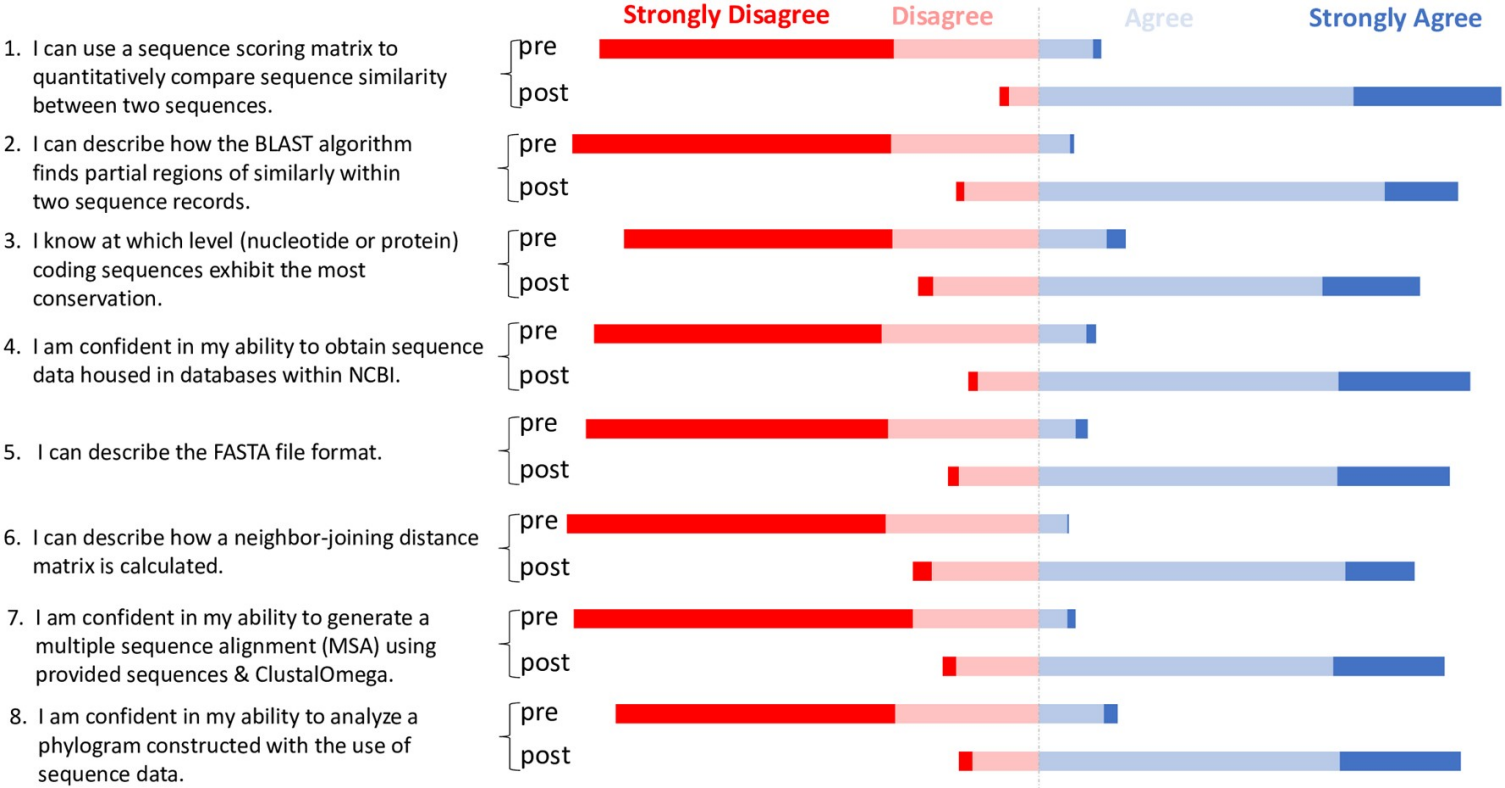
Student participants self-reported perceived learning gains. Retrospective pre- and post-survey aggregate data utilizing a four-point Likert-type scale is depicted as a divergent stacked bar graph. Nine cohorts of student participants (n = 362) at a diversity of institutions completed the survey instrument. All questions were statistically significant (p<0.0001) when comparing median Likert-type scale response between retrospective pre- and post-ratings using the Wilcoxon signed-rank test.

We evaluated whether self-reported student perceptions of their competence in targeted bioinformatics skills shifted after completing the sequence similarity learning modules. The items in the assessment instrument that probed perceived competence were used to answer this question. We looked at aggregate retrospective pre-/post-student perception of statements based on the module learning objectives (Fig 4). Aggregate data suggested that student participants (n = 362) retrospectively perceived they were not competent in module-associated concepts and skills before the intervention (a majority of responses were either ‘strongly disagree’ or ‘disagree’). However, after the intervention, a majority of students either ‘agreed’ or ‘strongly agreed’ that they were competent in the module-associated concepts and skills. This drastic shift in participant perceptions for each of the eight survey items was statistically significant (p<0.0001, Wilcoxon signed-rank test).

## Discussion

### A widely adaptable NIBLSE sequence similarity learning resource leads to measurable student learning gains

In this paper, we demonstrate that a bioinformatics resource that focuses on sequence similarity results in student learning gains. Collectively, assessment data showed objective student learning gains in both understanding and utilizing computational tools. Learning gains were found across classrooms, institutions, and student educational levels. That there were significant learning gains detected in upper-level classes suggests that bioinformatics integration across curricula is an ongoing process and reinforces the importance of producing and disseminating high-quality learning resources for life science educators.

Given the ad hoc recruitment of courses into the study, minimal consideration should be given to the differences among groups (e.g., course, institution type). While we observed a statistically significant increase in learning gains in courses taught at PUIs compared with RIs when considered as a group, the actual differences in learning gains among individual courses were small, and were reduced even further when we filtered the data to remove students whose time spent on the assessments indicated minimal effort. We expect that a multitude of difficult-to-control variables likely influenced the observed differences. These variables may include adaptations of the modules to fit specific course pedagogical goals or logistical constraints (e.g., in some cases streamlining the module to fit within the classroom period), instructional modality (e.g., face-to-face, distance instruction), amount of classroom time spent on the modules, variations in student/instructor interactions (e.g., frequency of interactions associated with class size, use of laboratory teaching assistants in some cases, rapport), and level of student (e.g., first-year, senior). These variables likely influenced the dataset and the resulting statistical analysis, but were difficult to isolate as having a notable effect. Although the collection of data from diverse courses complicated the analysis, the fact that significant learning gains were observed across the board is indicative that the learning modules are versatile, have utility in many types of courses, and at different academic levels. These results are consistent with other efforts to integrate an adaptable bioinformatics curriculum across diverse institutions [40].

A retrospective attitudinal survey indicated that students’ self-reported post-perception of competence in learning outcomes was significantly higher than their pre-perception, with medians on all questions shifting from negative to positive responses after module completion. The fact that students in a range of courses overwhelmingly indicated negative responses on the pre-survey perceptional items is further evidence of the need for a more concentrated effort to integrate bioinformatics into the life sciences; in particular, students taking upper-level courses did not report initial competence, suggesting that integration of bioinformatics into the first 1–2 years of undergraduate curricula is lacking. The perception by students of personal learning gains has been demonstrated to promote motivation and persistence within STEM fields, as self-efficacy is a requirement for persistence [24,41]. Helping students persevere is important across STEM fields and is critical to meet the increasing demand for biologists to have foundational knowledge of bioinformatics concepts and competencies as well as competent trainees going into emerging fields like bioinformatics [42].

The sequence similarity modules provide students with practice developing key data analysis skills, which are increasing in importance in contemporary research with an increased emphasis on computational data wrangling and analysis of big datasets resulting from wet-lab experiments [43]. Additionally, the modules allow students to experience the interdisciplinary nature of science, an AAAS *Vision and Change* core competency, by integrating concepts from molecular biology, evolution, computer science, statistics, and mathematics into a single exercise [4,44]. The modules, which rely on web-based computational tools, are easily adaptable resources independent of course modality (e.g., face-to-face, online instruction); indeed, two of our cohorts successfully implemented the modules in an asynchronous distance-learning environment. FMN members implemented the bioinformatics learning modules in a diverse array of courses, including AP Biology, Introductory Biology, Introductory Genetics, Conservation Genetics, Developmental Biology, Disease Ecology, Plant and Fungal Biology, Virology, and Bioinformatics. The modules were also readily adapted to fit specific course content with some of them shared publicly (e.g., botany, developmental biology, virology; [19–21]) in the NIBLSE resource collection available through QUBES. Additionally, the QUBES infrastructure provides a platform with a documented versioning process for iteratively updating the OER resource. The ability to update a resource serves to keep it up to date in a rapidly changing field. Additionally, others within the educational community can further adapt and share these modified module versions on QUBES along with detailed revision annotations. The set of modules with implementation instructions is also friendly to instructors with minimal bioinformatics experience looking to integrate bioinformatics principles into their introductory life science course for the first time. A majority of educators in our study with varied experience in bioinformatics successfully used these modules to introduce bioinformatics concepts for the first time in a diversity of courses, with support from the FMN for bioinformatics novices.

The originally published OER learning resource and its FMN adaptations continue to positively impact undergraduate life sciences education. Since the initial Incubator, the sequence similarity learning resource and its FMN adaptations have been accessed through the web >5,000 times and directly downloaded >1,500 times (S9 Table).

Here we harnessed a community-centered process to develop, implement, and assess a sequence similarity learning resource. This collaborative process allowed for the iterative development and validation of an assessment instrument coupled with the simultaneous collection of assessment data from varied classrooms. Assessment data were indicative of significant learning gains across diverse classrooms and implementation contexts. These data substantiate the value of this resource as a tool for the broad integration of bioinformatics competencies across undergraduate curricula.

## Supporting information

S1 Table Assessment questions aligned to learning resource learning content outcomes and the related NIBLSE core competencies (Wilson-Sayres et al., 2018) [7]. *NIBLSE Core Competencies 2 (Summarize key computational concepts, such as algorithms and relational databases, and their applications in the life sciences.), 4 (Use bioinformatics tools to examine complex biological problems in evolution, information flow, and other important areas of biology.), 5 (Find, retrieve, and organize various types of biological data.), and 8 (Describe and manage biological data types, structure, and reproducibility.).
(DOCX)

S2 Table Wilcoxon signed rank test for spring 2017 & 2018 200-level general biology course matched pre and retrospective pre-/post-student perceptions bioinformatics activity survey*. *n = 31, non-parametric Wilcoxon Signed Rank Test (two-tailed) with values represented as a median (typical analysis for ordinal data). P-values were independently calculated using the pre and retro pre with the post median and were <0.0001 for all tests.
(DOCX)

S3 Table Wilcoxon signed rank test for spring 2017 & 2018 300-level biotechnology course matched pre and retrospective pre-/post-student perceptions bioinformatics activity survey*. *n = 25, non-parametric Wilcoxon Signed-Rank Test (two-tailed) with values represented as a median (typical analysis for ordinal data). P-values were independently calculated using the pre and retro pre with the post median and were <0.0001 for all tests with the exceptions being the NCBI database (p = 0.0013) and Seq Conservation (p = 0.0002) questions with the true pre/post.
(DOCX)

S4 Table Post-assessment instrument item analysis*. *n = 373. Item Difficulty: #number of correct responses divided by the number of total responses. Item Discrimination: lower group (bottom 27%) percent correct subtracted from the upper group (top 27%) percent correct. Point-biserial correlation: correlation between score on an item and total score on the exam. Avg. Post—Avg. Pre: average pre-assessment score subtracted from average post-assessment score for each item.
(DOCX)

S5 Table Post-assessment instrument Cronbach’s Alpha reliability analysis*. *n = 373; overall Cronbach’s Alpha (ɑ) = 0.576; Cronbach’s Alpha if Item Deleted column represents the adjusted Cronbach’s Alpha if the indicated assessment item was excluded in the Cronbach’s Alpha calculation.
(DOCX)

S6 Table One-sample t-tests with Benjamini-Hochberg correction comparing course pre-/post-assessment score differences relative to zero. (DOCX)

S7 Table Two-factor generalized linear statistical models comparing pre-/post-assessment score differences and pre-assessment scores†. †Two factors: university type (Primarily Undergraduate Institution (PUI) vs. Research Intensive Institution (RI)) and course type. The base model is General Biology taught at a research-intensive institution. The intercept is associated with the base model and indicates the mean pre-/post- difference for the ’Difference in Pre-/Post-Assessment Score’ and the mean pre-score for the ’Pre-Assessment Score’ table sections, respectively. As indicated by the intercept, the base course exhibited significant learning gains; other RI courses had significantly higher estimates (p < 0.05) of average gains, while the average gains amongst PUI courses did not differ significantly (p > 0.05). SE = standard error; Significance, * = p<0.05, ** = p<0.01, *** = p<0.001. n = 373.
(DOCX)

S8 Table Two-factor generalized linear statistical model comparing pre-/post-assessment score differences on filtered dataset with pre-/post-records that took ≥4 minutes to complete†. †Two factors: university type (Primarily Undergraduate Institution vs. Research Intensive Institution) and course type. The base model is general biology taught at a research-intensive institution. The intercept is associated with the base model and indicates the mean difference in pre-/post- scores. SE = standard error; Significance, * = p<0.05, ** = p<0.01, *** = p<0.001. n = 306.
(DOCX)

S9 Table Direct open educational resource downloads and learning resource page views throughout the iterative process of revision and the generation of course-specific adaptations. * Learning resource adaptations created during the 2019 NIBLSE FMN.
(DOCX)

S1 Appendix Student assessment instruments (version 1). (DOCX)

S2 Appendix Student assessment instruments (version 2). (DOCX)

S1 Text Student participant pre-/post-assessment instrument development. (DOCX)
