# Supplementary material for: Community development, implementation, and assessment of a NIBLSE bioinformatics sequence similarity learning resource
Source: PLoS One. 2021 Sep 10;16(9):e0257404. doi: 10.1371/journal.pone.0257404 (PMC8432852; doi:10.1371/journal.pone.0257404)
Supplement: S1 Table — *NIBLSE Core Competencies 2 (Summarize key computational concepts, such as algorithms and relational databases, and their applications in the life sciences.), 4 (Use bioinformatics tools to examine complex biological problems in evolution, information flow, and other important areas of biology.), 5 (Find, retrieve, and organize various types of biological data.), and 8 (Describe and manage biological data types, structure, and reproducibility.). (DOCX) [file pone.0257404.s001.docx]

**S1 Table.** Assessment questions aligned to learning resource learning content outcomes and the related NIBLSE core competencies (Wilson-Sayres et al., 2018).

| **Lesson Learning Outcomes** | **NIBLSE Core Competency*** | **Associated Pre-/Post-Assessment Question(s)** |
| --- | --- | --- |
| Define similarity in a non-biological and biological sense when provided with two strings of letters. | 2 | 1, 2 |
| Quantify the similarity between two gene/protein sequences. | 2 | 3 |
| Explain how a substitution matrix is used to quantify similarity. | 2 | 4 |
| Calculate amino acid similarity scores using a scoring matrix. | 2 | 5 |
| Describe the simplified BLAST search algorithm including how similarity is used to perform a BLAST search. | 2 | 6, 7 |
| Demonstrate how to use bioinformatics tools to evaluate and analyze genomic data (e.g., BLASTP). | 4 | 8 |
| Create and interpret a nearest-neighbor distance matrix. | 4, 5, 8 | 9, 10 |
| Create a multiple sequence alignment using a nearest-neighbor distance matrix and a phylogram based on similarity of amino acid sequences. | 4, 5, 8 | 11, 12 |
| Distinguish between a rooted and unrooted phylogenetic tree. | 2 | 13 |
| Use appropriate bioinformatics sequence alignment tools and data format to investigate a biological question. | 4, 5, 8 | 14, 15 |

**NIBLSE Core Competencies 2 (Summarize key computational concepts, such as algorithms and relational databases, and their applications in the life sciences.), 4 (Use bioinformatics tools to examine complex biological problems in evolution, information flow, and other important areas of biology.), 5 (Find, retrieve, and organize various types of biological data.), and 8 (Describe and manage biological data types, structure, and reproducibility.).*
