# Supplementary material for: Community development, implementation, and assessment of a NIBLSE bioinformatics sequence similarity learning resource
Source: PLoS One. 2021 Sep 10;16(9):e0257404. doi: 10.1371/journal.pone.0257404 (PMC8432852; doi:10.1371/journal.pone.0257404)
Supplement: S2 Table — *n = 31, non-parametric Wilcoxon Signed Rank Test (two-tailed) with values represented as a median (typical analysis for ordinal data). P-values were independently calculated using the pre and retro pre with the post median and were <0.0001 for all tests. (DOCX) [file pone.0257404.s002.docx]

**S2 Table.** Wilcoxon Signed Rank Test for Spring 2017 & 2018 200-level general biology course matched Pre and Retrospective Pre-/Post-Student Perceptions Bioinformatics Activity Survey*

| **Item** | **Survey Item:** Strongly agree (5) to strongly disagree (1) | **Pre** Median | **Retro Pre** Median | **Post** Median | **P-value** |
| --- | --- | --- | --- | --- | --- |
| Scoring Matrix | I can use a sequence scoring matrix to quantitatively compare sequence similarity between two sequences. | 1 | 1 | 4 | <0.0001 |
| BLAST Algorithm | I can describe how the BLAST algorithm finds partial regions of similarity within two sequence records. | 1 | 1 | 4 | <0.0001 |
| Seq Conservation | I know at which level (nucleotide or protein) coding sequences exhibit the most conservation. | 2 | 1 | 4 | <0.0001 |
| NCBI Database | I am confident in my ability to obtain sequence data housed in databases within NCBI. | 1 | 1 | 4 | <0.0001 |
| FASTA Format | I can describe the FASTA file format. | 1 | 1 | 4 | <0.0001 |
| Distance Matrix | I can describe how a neighbor-joining distance matrix is calculated | 1 | 1 | 4 | <0.0001 |
| MSA Generation | I am confident in my ability to generate a multiple sequence alignment (MSA) using provided sequences and ClustalOmega | 1 | 1 | 4 | <0.0001 |
| Phylogram Analysis | I am confident in my ability to analyze a phylogram constructed with the use of sequence data. | 1 | 1 | 4 | <0.0001 |
| *n=31, non-parametric Wilcoxon Signed Rank Test (two-tailed) with values represented as a median (typical analysis for ordinal data). P-values were independently calculated using the pre and retro pre with the post median and were <0.0001 for all tests. | | | | | |
|  | | | | | |
