# Supplementary material for: Community development, implementation, and assessment of a NIBLSE bioinformatics sequence similarity learning resource
Source: PLoS One. 2021 Sep 10;16(9):e0257404. doi: 10.1371/journal.pone.0257404 (PMC8432852; doi:10.1371/journal.pone.0257404)
Supplement: S4 Table — *n = 373. Item Difficulty: #number of correct responses divided by the number of total responses. Item Discrimination: lower group (bottom 27%) percent correct subtracted from the upper group (top 27%) percent correct. Point-biserial correlation: correlation between score on an item and total score on the exam. Avg. Post—Avg. Pre: average pre-assessment score subtracted from average post-assessment score for each item. (DOCX) [file pone.0257404.s004.docx]

**S4 Table.** Post-Assessment Instrument Item Analysis. *

| **Item** | **Item Difficulty** | **Item Discrimination** | **Point-biserial correlation** | **Avg. Post - Avg Pre** |
| --- | --- | --- | --- | --- |
| 1 | 0.107 | 0.312 | 0.283 | 0.130 |
| 2 | 0.142 | 0.396 | 0.285 | 0.064 |
| 3 | 0.668 | 0.416 | 0.362 | 0.172 |
| 4 | 0.684 | 0.446 | 0.439 | 0.220 |
| 5 | 0.635 | 0.604 | 0.473 | 0.410 |
| 6 | 0.252 | 0.158 | 0.167 | -0.005 |
| 7 | 0.298 | 0.386 | 0.341 | 0.054 |
| 8 | 0.437 | 0.505 | 0.411 | 0.239 |
| 9 | 0.416 | 0.545 | 0.458 | 0.129 |
| 10 | 0.576 | 0.505 | 0.421 | 0.185 |
| 11 | 0.657 | 0.475 | 0.412 | 0.204 |
| 12 | 0.625 | 0.505 | 0.431 | 0.204 |
| 13 | 0.662 | 0.663 | 0.538 | 0.155 |
| 14 | 0.193 | 0.277 | 0.281 | 0.003 |
| 15 | 0.426 | 0.465 | 0.382 | 0.145 |

*n=373. Item Difficulty: #number of correct responses divided by the number of total responses. Item Discrimination: lower group (bottom 27%) percent correct subtracted from the upper group (top 27%) percent correct. Point-biserial correlation: correlation between score on an item and total score on the exam. Avg. Post - Avg. Pre: average pre-assessment score subtracted from average post-assessment score for each item.
