# Supplementary material for: Community development, implementation, and assessment of a NIBLSE bioinformatics sequence similarity learning resource
Source: PLoS One. 2021 Sep 10;16(9):e0257404. doi: 10.1371/journal.pone.0257404 (PMC8432852; doi:10.1371/journal.pone.0257404)
Supplement: S5 Table — *n = 373; overall Cronbach’s Alpha (ɑ) = 0.576; Cronbach’s Alpha if Item Deleted column represents the adjusted Cronbach’s Alpha if the indicated assessment item was excluded in the Cronbach’s Alpha calculation. (DOCX) [file pone.0257404.s005.docx]

**S5 Table.** Post-Assessment Instrument Cronbach's Alpha Reliability Analysis. *

| **Item** | **Scale Mean if Item Deleted** | **Scale Variance if Item Deleted** | **Corrected Item-Total Correlation** | **Cronbach's Alpha (ɑ) if Item Deleted** |
| --- | --- | --- | --- | --- |
| 1 | 6.670 | 6.919 | 0.120 | 0.575 |
| 2 | 6.635 | 6.852 | 0.077 | 0.590 |
| 3 | 6.110 | 6.707 | 0.198 | 0.562 |
| 4 | 6.094 | 6.517 | 0.286 | 0.546 |
| 5 | 6.142 | 6.406 | 0.318 | 0.539 |
| 6 | 6.525 | 6.208 | 0.007 | 0.593 |
| 7 | 6.480 | 6.773 | 0.181 | 0.565 |
| 8 | 6.340 | 6.549 | 0.243 | 0.553 |
| 9 | 6.362 | 6.426 | 0.297 | 0.543 |
| 10 | 6.201 | 6.525 | 0.255 | 0.551 |
| 11 | 6.121 | 6.573 | 0.252 | 0.552 |
| 12 | 6.153 | 6.510 | 0.270 | 0.548 |
| 13 | 6.115 | 6.251 | 0.396 | 0.525 |
| 14 | 6.584 | 6.965 | 0.140 | 0.571 |
| 15 | 6.351 | 6.630 | 0.211 | 0.560 |

*n=373; overall Cronbach's Alpha (ɑ) = 0.576; Cronbach's Alpha if Item Deleted column represents the adjusted Cronbach's Alpha if the indicated assessment item was excluded in the Cronbach's Alpha calculation.
