# Supplementary material for: Community development, implementation, and assessment of a NIBLSE bioinformatics sequence similarity learning resource
Source: PLoS One. 2021 Sep 10;16(9):e0257404. doi: 10.1371/journal.pone.0257404 (PMC8432852; doi:10.1371/journal.pone.0257404)
Supplement: S6 Table — (DOCX) [file pone.0257404.s006.docx]

**S6 Table. One-sample t-tests with Benjamini-Hochberg correction comparing course pre-/post-assessment score differences relative to zero.**

| **Course** | **n** | **statistic** | **p-value** | **adj. p-value** |
| --- | --- | --- | --- | --- |
| Gen Bio (RI) | 184 | 9.177986 | 0.00e+00 | 0.00e+00 |
| Bioinfo & Comp Bio (RI) | 31 | 7.358556 | 0.00e+00 | 1.00e-07 |
| Mol Bio (RI) | 76 | 8.632403 | 0.00e+00 | 0.00e+00 |
| Virology (RI) | 25 | 5.818182 | 5.30e-06 | 7.50e-06 |
| Gen Bio (PUI) | 20 | 6.490508 | 3.20e-06 | 5.60e-06 |
| Mol Biotech (PUI) | 23 | 4.185783 | 3.83e-04 | 3.83e-04 |
| Dev Bio (PUI) | 14 | 6.123317 | 3.64e-05 | 4.25e-05 |
