# Supplementary material for: Community development, implementation, and assessment of a NIBLSE bioinformatics sequence similarity learning resource
Source: PLoS One. 2021 Sep 10;16(9):e0257404. doi: 10.1371/journal.pone.0257404 (PMC8432852; doi:10.1371/journal.pone.0257404)
Supplement: S7 Table — †Two factors: university type (Primarily Undergraduate Institution (PUI) vs. Research Intensive Institution (RI)) and course type. The base model is General Biology taught at a research-intensive institution. The intercept is associated with the base model and indicates the mean pre-/post- difference for the ’Difference in Pre-/Post-Assessment Score’ and the mean pre-score for the ’Pre-Assessment Score’ table sections, respectively. As indicated by the intercept, the base course exhibited significant learning gains; other RI courses had significantly higher estimates (p < 0.05) of average gains, while the average gains amongst PUI courses did not differ significantly (p > 0.05). SE = standard error; Significance, * = p<0.05, ** = p<0.01, *** = p<0.001. n = 373. (DOCX) [file pone.0257404.s007.docx]

**S7 Table. Two-factor generalized linear statistical models comparing pre-/post-assessment score differences and pre-assessment scores. †**

| **Difference in Pre-/Post-Assessment Score (Post - Pre)** | | | | |  |
| --- | --- | --- | --- | --- | --- |
| **Coefficients** | **Estimate** | **SE** | **t-value** | **p-value** | **Significance** |
| Intercept | 1.7527 | 0.1889 | 9.280 | <0.00001 | *** |
| Institution | 1.7473 | 0.6032 | 2.897 | 0.00400 | ** |
| Course - Bioinformatics (RI) | 1.2634 | 0.4974 | 2.540 | 0.01150 | * |
| Course - Developmental Biology (PUI) | -0.4643 | 0.8928 | -0.520 | 0.60335 |  |
| Course - Molecular Biology (RI) | 0.8065 | 0.3493 | 2.309 | 0.02152 | * |
| Course - Molecular Biotechnology (PUI) | -1.0000 | 0.7833 | -1.277 | 0.20255 |  |
| Course - Virology (RI) | 1.4473 | 0.5461 | 2.650 | 0.00839 | ** |
| **Pre-Assessment Score** | | | | |  |
| **Coefficients** | **Estimate** | **SE** | **t-value** | **p-value** | **Significance** |
| Intercept | 4.1 | 0.49115 | 8.348 | <0.00001 | * |
| Institution | -0.08641 | 0.51715 | -0.167 | 0.86739 |  |
| Course - Bioinformatics (RI) | 0.95415 | 0.42644 | 2.237 | 0.02586 | * |
| Course - Developmental Biology (PUI) | 0.72143 | 0.7654 | 0.943 | 0.34653 |  |
| Course - Molecular Biology (RI) | 1.09826 | 0.2995 | 3.667 | 0.00282 | * |
| Course - Molecular Biotechnology (PUI) | -0.03478 | 0.67156 | -0.052 | 0.95872 |  |
| Course - Virology (RI) | 1.26641 | 0.46819 | 2.705 | 0.00715 | * |

†Two factors: university type (Primarily Undergraduate Institution (PUI) vs. Research Intensive Institution (RI)) and course type. The base model is General Biology taught at a research-intensive institution. The intercept is associated with the base model and indicates the mean pre-/post- difference for the 'Difference in Pre-/Post-Assessment Score' and the mean pre-score for the 'Pre-Assessment Score' table sections, respectively. As indicated by the intercept, the base course exhibited significant learning gains; other RI courses had significantly higher estimates (p < 0.05) of average gains, while the average gains amongst PUI courses did not differ significantly (p > 0.05). SE = standard error; Significance, * = p<0.05, ** = p<0.01, *** = p<0.001. n=373.
