# Supplementary material for: Community development, implementation, and assessment of a NIBLSE bioinformatics sequence similarity learning resource
Source: PLoS One. 2021 Sep 10;16(9):e0257404. doi: 10.1371/journal.pone.0257404 (PMC8432852; doi:10.1371/journal.pone.0257404)
Supplement: S8 Table — †Two factors: university type (Primarily Undergraduate Institution vs. Research Intensive Institution) and course type. The base model is general biology taught at a research-intensive institution. The intercept is associated with the base model and indicates the mean difference in pre-/post- scores. SE = standard error; Significance, * = p<0.05, ** = p<0.01, *** = p<0.001. n = 306. (DOCX) [file pone.0257404.s008.docx]

**S8 Table.** Two-factor generalized linear statistical model comparing pre-/post-assessment score differences on filtered dataset with pre-/post-records that took ≥4 minutes to complete.†

| **Difference in Pre-/Post-Assessment Score (Post - Pre)** | | | | |  |
| --- | --- | --- | --- | --- | --- |
| **Coefficients** | **Estimate** | **SE** | **t-value** | **p-value** | **Significance** |
| Intercept | 2.0573 | 0.2220 | 9.268 | <0.00001 | *** |
| Institution | 1.4427 | 0.6099 | 2.365 | 0.01860 | * |
| Course - Bioinformatics | 1.2463 | 0.5290 | 2.356 | 0.01910 | * |
| Course - Developmental Biology | -0.4643 | 0.8853 | -0.524 | 0.60040 |  |
| Course - Molecular Biology | 0.6442 | 0.3816 | 1.688 | 0.09240 |  |
| Course - Molecular Biotechnology | -1.0682 | 0.7849 | -1.361 | 0.17460 |  |
| Course - Virology | 0.9427 | 0.5641 | 1.671 | 0.09570 |  |

†Two factors: university type (Primarily Undergraduate Institution vs. Research Intensive Institution) and course type. The base model is general biology taught at a research-intensive institution. The intercept is associated with the base model and indicates the mean difference in pre-/post- scores. SE = standard error; Significance, * = p<0.05, ** = p<0.01, *** = p<0.001.  n=306.
