# Supplementary material for: Community development, implementation, and assessment of a NIBLSE bioinformatics sequence similarity learning resource
Source: PLoS One. 2021 Sep 10;16(9):e0257404. doi: 10.1371/journal.pone.0257404 (PMC8432852; doi:10.1371/journal.pone.0257404)
Supplement: S9 Table — * Learning resource adaptations created during the 2019 NIBLSE FMN. (DOCX) [file pone.0257404.s009.docx]

**S9 Table**. Direct open educational resource downloads and learning resource page views throughout the iterative process of revision and the generation of course-specific adaptations.

| **Learning Resource Version or Adaptation** | **Views (Downloads) as of 1/15/2021** |
| --- | --- |
| Sequence Similarity v 1.0 - QUBES | 539 (103) |
| Sequence Similarity v 2.0 - QUBES | 220 (48) |
| Sequence Similarity v 3.0 - QUBES | 288 (45) |
| Sequence Similarity v 4.0 - QUBES | 1100 (226) |
| Sequence Similarity v 5.0 - QUBES | 1194 (181) |
| Sequence Similarity – *CouseSource* Published Supporting Materials | N/A (139) |
| Sequence Similarity in Developmental Biology - A Bioinformatics Exercise Using Myostatin v 1.0 - QUBES * | 282 (143) |
| Bioinformatics: Investigating Sequence Similarity - A Plant Biology Approach v 1.0 - QUBES * | 796 (286) |
| Sequence Similarity Resource Adaptation: Exploring Ebola Virus v 1.0 - QUBES * | 658 (333) |
| **Total** | **5,077 (1,504)** |

* Learning resource adaptations created during the 2019 NIBLSE FMN
