# Supplementary material for: Community development, implementation, and assessment of a NIBLSE bioinformatics sequence similarity learning resource
Source: PLoS One. 2021 Sep 10;16(9):e0257404. doi: 10.1371/journal.pone.0257404 (PMC8432852; doi:10.1371/journal.pone.0257404)
Supplement: S1 Appendix — (DOCX) [file pone.0257404.s010.docx]

**S1 Appendix.** Student Assessment Instruments (Version 1).

*For each of the items below, circle the Likert scale response that best characterizes how you feel about the statement, where: 1 = Strongly Disagree, 2 = Disagree, 3 = Neutral, 4 = Agree, 5 = Strongly Agree.*

|  | Strongly Disagree | Disagree | Neutral | Agree | Strongly Agree |
| --- | --- | --- | --- | --- | --- |
| 1. I have been previously exposed to the topics of sequence similarity and bioinformatics. | 1 | 2 | 3 | 4 | 5 |
| 1. I have been previously exposed to the topic of molecular phylograms. | 1 | 2 | 3 | 4 | 5 |
| 1. I can use a sequence scoring matrix to quantitatively compare sequence similarity between two sequences. | 1 | 2 | 3 | 4 | 5 |
| 1. I can describe how the BLAST algorithm finds partial regions of similarly within two sequence records. | 1 | 2 | 3 | 4 | 5 |
| 1. I know at which level (nucleotide or protein) coding sequences exhibit the most conservation. | 1 | 2 | 3 | 4 | 5 |
| 1. I am confident in my ability to obtain sequence data housed in databases within NCBI. | 1 | 2 | 3 | 4 | 5 |
| 1. I can describe the FASTA file format. | 1 | 2 | 3 | 4 | 5 |
| 1. I can describe how a neighbor-joining distance matrix is calculated. | 1 | 2 | 3 | 4 | 5 |
| 1. I am confident in my ability to generate a multiple sequence alignment (MSA) using provided sequences and ClustalOmega. | 1 | 2 | 3 | 4 | 5 |
| 1. I am confident in my ability to analyze a phylogram constructed with the use of sequence data. | 1 | 2 | 3 | 4 | 5 |

**Student Post-Assessment Instrument (Version 1)**

*For each of the items below, circle the Likert scale response that best characterizes how you feel about the statement, where: 1 = Strongly Disagree, 2 = Disagree, 3 = Neutral, 4 = Agree, 5 = Strongly Agree.*

|  | **Your Perception BEFORE Starting the Bioinformatics Modules** | | | | | **Your Perception AFTER Completing the Bioinformatics Modules** | | | | |
| --- | --- | --- | --- | --- | --- | --- | --- | --- | --- | --- |
|  | Strongly Disagree | Disagree | Neutral | Agree | Strongly Agree | Strongly Disagree | Disagree | Neutral | Agree | Strongly Agree |
| 1. I can use a sequence scoring matrix to quantitatively compare sequence similarity between two sequences. | 1 | 2 | 3 | 4 | 5 | 1 | 2 | 3 | 4 | 5 |
| 1. I can describe how the BLAST algorithm finds partial regions of similarly within two sequence records. | 1 | 2 | 3 | 4 | 5 | 1 | 2 | 3 | 4 | 5 |
| 1. I know at which level (nucleotide or protein) coding sequences exhibit the most conservation. | 1 | 2 | 3 | 4 | 5 | 1 | 2 | 3 | 4 | 5 |
| 1. I am confident in my ability to obtain sequence data housed in databases within NCBI. | 1 | 2 | 3 | 4 | 5 | 1 | 2 | 3 | 4 | 5 |
| 1. I can describe the FASTA file format. | 1 | 2 | 3 | 4 | 5 | 1 | 2 | 3 | 4 | 5 |
| 1. I can describe how a neighbor-joining distance matrix is calculated. | 1 | 2 | 3 | 4 | 5 | 1 | 2 | 3 | 4 | 5 |
| 1. I am confident in my ability to generate a multiple sequence alignment (MSA) using provided sequences and ClustalOmega. | 1 | 2 | 3 | 4 | 5 | 1 | 2 | 3 | 4 | 5 |
| 1. I am confident in my ability to analyze a phylogram constructed with the use of sequence data. | 1 | 2 | 3 | 4 | 5 | 1 | 2 | 3 | 4 | 5 |

1. What did you enjoy about the activity?
2. Was there anything that you did not enjoy about the activity?
3. Can you think of any way the activity could be improved?
4. General Comments about the activity or the above questions:
5. Briefly explain how a biologist would obtain the total similarity score between two homologous protein sequences of the same length.
6. Why would a biologist use the BLAST algorithm to look for sequence similarity between a query sequence and a set of database sequence records rather than directly aligning the query to each database record?
7. Discuss the advantages and disadvantages of using a nucleotide vs. a protein sequence when looking for a homologous gene in other species within a bioinformatics database?
8. Describe the FASTA sequence format.
9. What does branch length represent in a molecular phylogram? Explain.

**Summative Post-Activity Assessment Questions**

| **DIMENSIONS** | **Associated Student Learning Outcomes** | **NIBLSE Core Competency *** | **EXEMPLARY (2)** | **BEGINNER (1)** | **UNSATISFACTORY (0)** |
| --- | --- | --- | --- | --- | --- |
| **1.**Briefly explain how a biologist would obtain a similarity score between two homologous protein sequences of the same length. | 2, 3 | #2 | Student states the sequences should be **aligned end-to-end** (may be assumed based on description of matrix usage) and then the alignment quantified using a **substitution matrix.** | Student indicates the sequences should be aligned, however **makes a claim that is false** and/or does not name/describe a substitution matrix (assumes use of identity or lacks clarity). | Student provides incorrect ideas on how to determine similarity via alignment.  Does not answer question |
| **2.** Why would a biologist use the BLAST algorithm to look for sequence similarity between a query sequence and a set of database sequence records rather than directly aligning the query to each database record? | 5, 6 | #2 | Student states to reduce computational cost (**time**), may elaborate on the algorithm breaking the seq into words. | Student states to reduce time, however, **makes a claim that is false or lacks clarity**. | Student provides incorrect ideas or  Does not answer question |
| **3.** Discuss the advantages and disadvantages of using nucleotide vs. protein sequences when looking for a homologous gene in other species within a bioinformatics database? | 2 | #4 | Student provides an accurate rationale for use of nucleotide vs. proteins sequences (degree of conservation, degeneracy of genetic code) | Student provides a somewhat accurate rationale for use, but it might not be clear or has a false statement | Student response lacks an accurate reason why a research would choose one over the other  Does not answer question |
| **4.** Describe the FASTA sequence format. | 8 | #8 | Student defines at least two of the three components: (1) first line is = > description and (2) second/subsequent lines (seq). (3) text-based format | Student defines one or two components of FASTA format and/or has a false statement. Does not state that format is only nucleotide or protein sequences. | Student provides incorrect ideas or  Does not answer question |
| **5.** What does branch length represent in a molecular phylogram? Explain. | 9 | #4 | Student states the relationship between distance and sequence changes (evo time) for a specific genetic locus. | Student identifies that > distance associated with > time, but does not state seq changes (assumes whole organism) and/or false statement | Student provides incorrect ideas or  Does not answer question |

*[*https://qubeshub.org/community/groups/niblse/core_competencies*](https://qubeshub.org/community/groups/niblse/core_competencies)
