# Supplementary material for: Community development, implementation, and assessment of a NIBLSE bioinformatics sequence similarity learning resource
Source: PLoS One. 2021 Sep 10;16(9):e0257404. doi: 10.1371/journal.pone.0257404 (PMC8432852; doi:10.1371/journal.pone.0257404)
Supplement: S2 Appendix — (DOCX) [file pone.0257404.s011.docx]

**S2 Appendix.** Student Assessment Instruments (Version 2).

1. Which of the following **BEST** represents how to quantitate similarity between the following two phrases. Select **ALL** that apply.

*“The goat jumped over the bridge”* and *“The rabbit hopped over the fence”*

- **Align the phrases and reward identical words and penalize non-identical words with numerical values.**
- **Align the phrases and do not reward or penalize words that differ but have a similar meaning with numerical values.**
- Align the phrases and generate a numerical value (0 or 1) based on if the phrases are identical or not.
- Align the phrases and generate a categorical system of “dissimilar”, “somewhat similar, “very similar”, and “identical” based on intuition.

1. Which of the following statements regarding homology and/or significant sequence similarity is/are correct. Select **ALL** that apply.

- All complex sequences that exhibit significant similarity in a pairwise sequence comparison are homologous.
- All homologous sequences exhibit significant similarity in a pairwise sequence comparison.
- **Complex sequences that exhibit significant similarity in a pairwise sequence comparison are not always homologous.**
- **Homologous sequences do not always exhibit significant similarity in a pairwise sequence comparison.**

1. Which of the following describes how a biologist would obtain an overall similarity score between two homologous protein sequences of the same length.
2. **Align the two proteins and calculate a similarity score using a BLOSUM scoring matrix for each aligned residue.**
3. Align the two proteins and calculate a similarity score by dividing the number of identities by the total number of amino acids in the sequence.
4. Align the two proteins and calculate a similarity score by dividing the number of non-identities by the total number of amino acids in the sequence.
5. Align both proteins to themselves to generate the maximum alignment score and multiply the maximum value by the average rate of evolutionary change between the two species the sequences were derived from.
6. Which of the following describes why some amino acid substitutions within a BLOSUM substitution matrix are scored positively, while other substitutions are scored negatively.
   - **The substitution score is influenced by the chemical properties and size of the substituted amino acid residue with similar chemical properties and size potentially providing a positive substitution score.**
   - Substitutions at the protein level are expected and may not influence structure since the genetic code is degenerate, thus leading to a positive substitution score.
   - A small subset of the 20 commonly occurring amino acids possess small functional groups that do not greatly influence protein folding and thus reflect a positive substitution score no matter how similar they are to the substituted residue.
   - Select substitutions that result in replacing a large bulky ring structured R-group with a smaller more flexible functional R-group allow some proteins to better interact with other molecules and facilitate induced fit interactions.
7. Calculate the similarity score for the following two peptides using the BLOSUM scoring matrix below.

Peptides: “QLN” and “MNN”


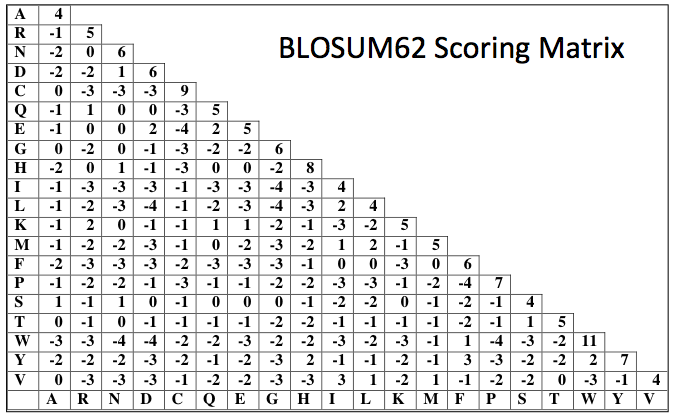


- 1. -1
  2. -2
  3. -3
  4. -6
  5. 1
  6. 2
  7. **3**
  8. 6

1. Which of the following **DOES NOT** describe a step taken during execution of the BLAST algorithm with a protein sequence.
   1. A query sequence is cut into smaller query words that will be aligned to all of the databases sequences.
   2. A BLOSUM scoring matrix is used to score alignments and these scores are reported on the results page.
   3. Initial BLAST alignments are extended in both directions until the score of the aligned residues hits a cutoff threshold.
   4. **Portions of the query sequence undergo a multiple sequence alignment with database records to streamline the algorithm’s computational costs.**
2. Which of the following describes why a biologist would use the BLAST algorithm to look for sequence similarity between a query sequence and a set of database sequence records rather than using an algorithm to directly aligning the query to each database record?
3. Different sizes in query and database records may make it hard to align the sequences.
4. **BLAST takes less computational time by breaking a sequence into shorter “words” to seed an alignment prior to extending to the full alignment.**
5. BLAST takes less computational time by executing a multiple sequence alignment, which filters out sequences from unrelated species and aligns the full length query to a smaller subset of sequences.
6. The BLAST algorithm generates a phylogram as output that makes it easy to quickly compare sequences visually.
7. Which of the following **BEST** describes sound reasoning behind choosing a nucleotide vs. protein sequence for identifying homologous genes in other distantly related species within a bioinformatics database?
8. **PROTEIN sequences are preferred because they are more highly conserved compared to nucleotide sequences of the same coding region because the genetic code is degenerate.**
9. NUCLEOTIDE sequences are preferred because they are more highly conserved compared to protein sequences of the same coding region because the genetic code is degenerate.
10. NUCLEOTIDE sequences are preferred for searching for potential homologs since nucleotide databases are larger and more encompassing.
11. PROTEIN sequences are preferred for searching for potential homologs since protein databases are larger and more encompassing.
12. The phylogram below provides a visual of the relationship between the sequences associated with a molecular clock gene from four separate species. Refer to the distance matrix and the corresponding phylogram to infer the identity of species “4” within the phylogram.


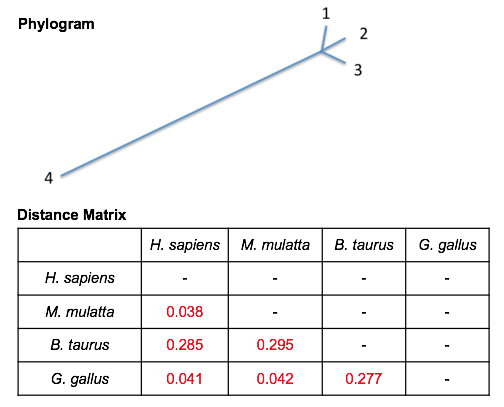


- 1. *H. sapiens*
  2. *M. mulatta*
  3. ***B. taurus***
  4. *G. gallus*

1. Using the data within the following distance matrix, determine which two species should be joined during the first round of neighbor joining when generating a phylogram.


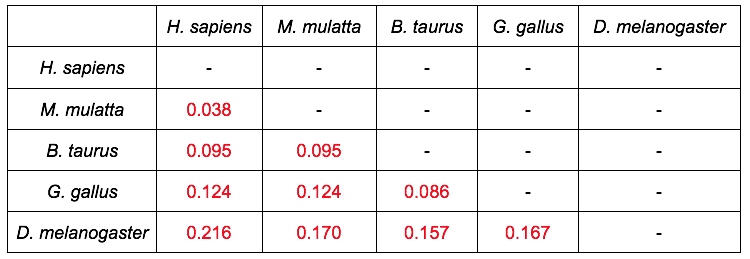


- 1. ***H. sapiens* and *M. mulatta***
  2. *H. sapiens* and *D. melanogaster*
  3. *D. melanogaster* and *G. gallus*
  4. *B. taurus* and *G. gallus*

1. Which of the following best describes what branch length represents within a molecular phylogram.
2. Branch length represents the GENETIC distance between sequences, which can be used to infer evolutionary relationships, the shorter the branch the GREATER the number of changes.
3. **Branch length represents the GENETIC distance between sequences, which can be used to infer evolutionary relationships, the shorter the branch the FEWER the number of changes.**
4. Branch length represents the MORPHOLOGICAL distance between sequence-source organisms, which can be used to infer evolutionary relationships, the shorter the branch the GREATER the number of changes.
5. Branch length represents the MORPHOLOGICAL distance between sequence-source organisms, which can be used to infer evolutionary relationships, the shorter the branch the FEWER the number of changes.
6. Which of the following would be the first step in generating a phylogram from a group of biological sequences.
   1. **Multiple sequence alignment**
   2. Generation of a distance matrix
   3. Neighbor joining
   4. Tree evaluation
7. Choose the **MOST APPROPRIATE** scenario for when a biologist would want to use an unrooted phylogram opposed to a rooted phylogram to demonstrate relationships regarding sequence similarity.
   1. **When a known common ancestor of the sequences has not been identified to be used as an outgroup.**
   2. When the user would like to generate a tree topology that provides an abstract shape.
   3. When a phylogram has more than six NODES with variable branch lengths.
   4. When a phylogram has more than six CLADES with variable branch lengths.
8. You have obtained a molecular clock gene protein sequence for what appears to be a novel species of iguana, along with a set of reptile protein reference sequences. Which of the following describes an appropriate approach to determine which reference species your unknown sequence has the most sequence similarity to.
   1. **Perform a multiple sequence alignment with the protein sequences and generate a phylogram.**
   2. Perform a pairwise alignment between each reference sequence using a BLOSUM scoring matrix and to identify the pair with the lowest score, which is associated with the fewest substitutions.
   3. Perform a BLAST search on a non-redundant protein database and create a phylogram with the first set of returned hits.
   4. Perform an NCBI database search for the reference sequences to track down the associated nucleotide sequences and execute a multiple sequence alignment followed by generating a phylogram.
9. Which of the following **BEST** describes FASTA sequence format.
10. **Represents sequence of nucleotides OR amino acids. Includes a sequence description line following a greater than sign and sequence data on subsequent lines.**
11. Represents sequence of amino acids ONLY. Includes a sequence description line following a greater than sign and sequence data on subsequent lines.
12. Represents sequence of nucleotides OR amino acids. Includes a formatted sequence description that needs to be modified for the program using the data and sequence data on subsequent lines.
13. Represents sequence of amino acids ONLY. Includes a formatted sequence description that needs to be modified for the program using the data and sequence data on subsequent lines.

*For each of the questions below, circle the Likert scale response that best characterizes how you feel about the statement, where: 1 = Strongly Disagree, 2 = Disagree, 3 = Agree, 4 = Strongly Agree.*

|  | **Your Perception BEFORE Starting the Bioinformatics Modules** | | | | **Your Perception AFTER Completing the Bioinformatics Modules** | | | |
| --- | --- | --- | --- | --- | --- | --- | --- | --- |
|  | Strongly Disagree | Disagree | Agree | Strongly Agree | Strongly Disagree | Disagree | Agree | Strongly Agree |
| 1. I can use a sequence scoring matrix to quantitatively compare sequence similarity between two sequences. | 1 | 2 | 3 | 4 | 1 | 2 | 3 | 4 |
| 1. I can describe how the BLAST algorithm finds partial regions of similarity within two sequence records. | 1 | 2 | 3 | 4 | 1 | 2 | 3 | 4 |
| 1. I know at which level (nucleotide or protein) coding sequences exhibit the most conservation. | 1 | 2 | 3 | 4 | 1 | 2 | 3 | 4 |
| 1. I am confident in my ability to obtain sequence data housed in databases within NCBI. | 1 | 2 | 3 | 4 | 1 | 2 | 3 | 4 |
| 1. I can describe the FASTA file format. | 1 | 2 | 3 | 4 | 1 | 2 | 3 | 4 |
| 1. I can describe how a neighbor-joining distance matrix is calculated. | 1 | 2 | 3 | 4 | 1 | 2 | 3 | 4 |
| 1. I am confident in my ability to generate a multiple sequence alignment (MSA) using provided sequences and ClustalOmega. | 1 | 2 | 3 | 4 | 1 | 2 | 3 | 4 |
| 1. I am confident in my ability to analyze a phylogram constructed with the use of sequence data. | 1 | 2 | 3 | 4 | 1 | 2 | 3 | 4 |
