# Supplementary material for: Community development, implementation, and assessment of a NIBLSE bioinformatics sequence similarity learning resource
Source: PLoS One. 2021 Sep 10;16(9):e0257404. doi: 10.1371/journal.pone.0257404 (PMC8432852; doi:10.1371/journal.pone.0257404)
Supplement: S1 Text — (DOCX) [file pone.0257404.s012.docx]

**S1 Text.** Student Participant Pre-/Post-Assessment Instrument Development.

## Assessment Instrument Version 1 Pilots

Between the NIBLSE incubator and NIBLSE FMN version 1 of the assessment instrument and student perceptions survey (see S1 Appendix) was administered during the Spring of 2017 and 2018 in a 200-level biology course and a 300-level biology course at independent primarily undergraduate institutions. The post-survey was designed with 5-point retrospective pre-Likert items nested adjacent to the post-Likert items to control for response-shift bias (Howard et al., 1979). In other words, participants were asked to reflect on their memory of knowledge prior to the intervention and their current knowledge state after the intervention to control for bias such as experience limitations. True pre-survey data with the same Likert-style items were used as a control mechanism due to students' tendency to inflate their perceived understanding of future content on a pretest (Aiken & West, 1990). We observed this phenomenon with both pilot implementing institutions with a more frequent response-shift bias detected in participants enrolled in the 300-level biology course (S2-S3 Tables).

## Assessment Instrument Version 2 Reliability Analysis

Further analysis showed that ɑ could be increased to 0.593 by removing Item #6 or to 0.590 by removing Item #2 (S5 Table), consistent with the point-biserial analysis. Those two items also show problematic discrimination.  Since Cronbach's Alpha statistics suggested that a slight improvement in assessment reliability might be achieved by sequentially removing particular items, that approach was investigated further. When removing items #2 and #6, Cronbach's Alpha reliability rose above the 0.6 threshold to 0.609. This would suggest when an instructor tailors the assessment to their own classroom instruction, they should focus on items #2 and #6 for assessment refinements in association with their own instruction or content emphasis, or remove one or both of these items if they are not aligned to classroom learning outcomes. It is also important to note that ɑ, although certainly useful and common for investigating the reliability of concept-focused assessments for classroom instruction like this one, does tend to underestimate the reliability of an assessment when scales differ across some questions and when multiple factors or traits underlie the items (Tavakol and Dennick, 2011). As our questions strive to reach across various interrelated concepts associated with student understanding of bioinformatics, an underestimate by ɑ would be expected.
